# Mixed response and time-to-event endpoints for multistage single-arm phase II design

**DOI:** 10.1186/s13063-015-0743-9

**Published:** 2015-06-04

**Authors:** Xin Lai, Benny Chung-Ying Zee

**Affiliations:** Division of Biostatistics, Jockey Club School of Public Health and Primary Care, Room 501, JC School of Public Health and Primary Care, Prince of Wales Hospital, The Chinese University of Hong Kong, Shatin, Hong Kong; Clinical Trials and Biostatistics Lab, Shenzhen Research Institute, The Chinese University of Hong Kong, Shenzhen, China

**Keywords:** Phase II trial design, Multiple-endpoints, Cytostatic drug testing, Target therapy

## Abstract

**Background:**

The objective of phase II cancer clinical trials is to determine if a treatment has sufficient activity to warrant further study. The efficiency of a conventional phase II trial design has been the object of considerable debate, particularly when the study regimen is characteristically cytostatic. At the time of development of a phase II cancer trial, we accumulated clinical experience regarding the time to progression (TTP) for similar classes of drugs and for standard therapy. By considering the time to event (TTE) in addition to the tumor response endpoint, a mixed-endpoint phase II design may increase the efficiency and ability of selecting promising cytotoxic and cytostatic agents for further development.

**Methods:**

We proposed a single-arm phase II trial design by extending the Zee multinomial method to fully use mixed endpoints with tumor response and the TTE. In this design, the dependence between the probability of response and the TTE outcome is modeled through a Gaussian copula.

**Results:**

Given the type I and type II errors and the hypothesis as defined by the response rate (RR) and median TTE, such as median TTP, the decision rules for a two-stage phase II trial design can be generated. We demonstrated through simulation that the proposed design has a smaller expected sample size and higher early stopping probability under the null hypothesis than designs based on a single-response endpoint or a single TTE endpoint.

**Conclusions:**

The proposed design is more efficient for screening new cytotoxic or cytostatic agents and less likely to miss an effective agent than the alternative single-arm design.

**Electronic supplementary material:**

The online version of this article (doi:10.1186/s13063-015-0743-9) contains supplementary material, which is available to authorized users.

## Background

The primary objective of phase II trials in oncology is to identify the agents or treatments that are sufficiently efficacious in antitumor activity to warrant further investigation in phase III trials. The tumor response rate (RR) is a common primary endpoint used to indicate possible antitumor activity for a study treatment in phase II cancer clinical trials [1]. However, studies of a few novel agents in recent years have revealed that other endpoints, such as the time to progression (TTP) or progression-free survival (PFS), are also relevant in assessing the antitumor activity of various new agents [2–5]; this is because several of the studied agents have been reported to prolong the TTP or PFS instead of improving tumor RR [6, 7]. For example, despite a low tumor RR [8], agents such as sorafenib in renal cell carcinoma have been observed to have significant PFS and overall survival benefits [6]. Therefore, relying on a single traditional RR can lead to an unexpectedly high type II error, meaning that promising drugs are likely to be missed because of a lack of observed activity.

Phase II trials can also be used to rapidly terminate inefficacious drugs that do not warrant further development. The multistage design, which is typically a two-stage design, was developed to screen out inactive drugs at the interim stages. One of the advantages of this method is that it enables early termination of a futile study and consequently patient resources can be conserved for other studies. Fleming [9] and Simon [10] proposed multistage designs where tumor RR is the only endpoint in assessing antitumor activity for drugs. Although phase II designs based on TTP or PFS endpoints have received considerable attention in the past decade [11–13], using the time-to-event (TTE) endpoint alone requires a longer period for assessing the outcome, which may not be an ideal screening tool for selecting active drugs and terminating inactive drugs [14]. In some clinical trials, a new therapeutic agent at the time of phase II development might have uncertain levels of drug activity, regardless of the extent to which it has been studied, and whether the TTE endpoint alone is the optimal choice is unclear. Therefore, combining both the response endpoint and the TTE endpoint for assessing new agents is a logical option. For example, in a Phase II study of antisense AEG35156 in combination with sorafenib for advanced hepatocellular carcinoma (HCC), the original design was to use TTP alone as primary endpoint because the drug activity of this antisense was expected to improve TTP more so than tumor RR. However, the benefits based on TTP were not obvious enough to show drug activity but tumor RR did. The results have shown that the median TTP was 4.0 months and 2.6 months for the study treatment and control arms, respectively. The primary TTP was in favor of the study treatment but did not reach statistical significance since the sample size was designed to be small in this Phase II study. It was further pointed out that patients who had dose modifications according to protocol did significantly better in TTP than those who had no dose reduction, possibly due to potential side effects. The response status as an outcome showing short term drug activity may also contribute, and in this example we have observed a clear treatment activity in the study treatment and no activity in the control (5 versus 0 responses). If we had used a mixed endpoints design in the first place, we would have shown the drug was active in this study [15].

Zee et al. [16] and Sun et al. [17] proposed a multinomial design to accommodate both tumor response and progressive disease in evaluating the effectiveness of a study agent, in which the early progressive disease (EPD) rate was incorporated into the composite hypothesis setting. The additional information from the EPD endpoint enabled the multinomial design to provide a better decision rule than those based on the response endpoint alone, with a higher probability of early stopping and smaller expected sample size [18]. However, the lack of concordance between binary EPD at a fixed time point in phase II trials and TTE endpoints such as TTP, PFS, and overall survival (OS) in subsequent phase III studies suggests that EPD may not be the most appropriate endpoint for developing a multistage phase II design [5]. The discrete characteristic of the EPD endpoint may lose crucial information because of its arbitrary definition in the choice of a fixed time point in the evaluation [19].

In this study, we considered using the tumor RR and a TTE endpoint such as TTP or PFS, instead of the dichotomized EPD variable, for developing a stopping rule for multistage single-arm phase II trials. Because of the association between TTE and RR endpoints within the same patient, and because ignoring such an association can lead to higher type I or type II errors, we adopted the Gaussian copula method to model the dependence structure between a binary RR endpoint and a continuous TTE endpoint. If the tumor response probability is determined by a normal variable through the probit model and the underlying TTE is assumed to follow an exponential distribution, then the dependence between RR and TTE is expressed as a correlation between the underlying normal variable and the exponential variable. Under these conditions, our design allows early rejection of drugs if they have an unacceptably low RR after stage I and a short median TTE. The Methods section describes the multistage hypothesis-testing procedure based on the copula model. The Results section reports a simulation study conducted to assess the performance of the proposed design under various correlation settings.

## Methods

In our phase II clinical trial design with tumor response and TTE endpoints, the null hypothesis and the alternative hypothesis are expressed as

1 $$ {H}_0:\ \left(p\le {p}_0\ \mathrm{and}\ {T}_{med}^{*}\le {T}_0\right)\kern1em \mathrm{versus}\kern0.75em {H}_1:\ \left(p>{p}_1\ \mathrm{or}\ {T}_{med}^{*}>{T}_1\right) $$

where *T*_med_^*^ is the true median TTE T* that is assumed to follow exponential distribution with hazard rate *λ* and hence *T*_med_^*^ = ln 2/*λ*. Expecting that a correlation between tumor response endpoint and TTE endpoint exists is logical because a high RR is typically related to a long TTE, particularly in studies with cytotoxic agents [20]. Therefore, in this design, the dependence between the probability of response and the hazard rate function for the TTE endpoint is modeled using a Gaussian copula (Appendix section A). We further assumed that the censoring time *T*_*i*_^*C*^ is noninformative (i.e.*,* the marginal density function of tumor response and the true TTE and the dependence structure are not affected by censoring once the copula is prespecified in the design). In practice, the censoring observations in most trials affect the true median TTE. Therefore, for the null hypothesis that experimental treatment is inactive, the decision to reject the null hypothesis can be made based on the Kaplan–Meier median *T*_*med*_ derived from the observed TTE min{*T*_i_^*^, *T*_*i*_^*C*^}, *i* = 1, … which is consistent with the true median TTE in distribution [21], as well as the total number of tumor responses ∑_i = 1_^N^Y_i_ where Y_i_ is the tumor response indicator of the *i*th patient (Appendix). However, deriving the analytical form of the joint distribution of these two statistics by using the copula structure is not possible. Hence, we used a simulation-based approach (Appendix section B) to specify the critical values. The censoring time was generated independently from an exponential distribution with hazard rate *λ*^*C*^ to obtain the observed time because noninformative censoring was assumed in the design. To achieve the predetermined censoring rate *r*^*C*^ for the TTE, the censoring hazard rate was set as *λ*^*C*^ = *λr*^*C*^/(1 − *r*^*C*^), which implies that the hazard rate *λ*^*C*^ in generating early stopping rules for futility (H_1_) differs from that for activity (H_0_). For simplicity, we considered the censoring rates in both H_0_ and H_1_ to be the same, although the design allows distinct censoring for H_0_ and H_1_. Because the correlation coefficient *ρ* specified in copula (Appendix section A) influences the decision boundary, the simulation-based method was adopted under a different correlation setting to obtain the appropriate decision criterion. We assumed a positive correlation because a high RR is likely to be associated with a long median TTE regarding TTP or PFS, when treatment is expected to be active.

In many clinical trials, most investigators prefer to continue the study to improve the estimation accuracy at the interim analysis, even if early rejection criteria of the null hypothesis are fulfilled. Therefore, we considered only early stopping for futility in the proposed design. We developed an R computer program for determining the phase II stopping criteria for the proposed method. Users need to specify the parameters for the hypotheses, the nominal type I and type II errors, and a fixed sample size for stage I and the final stage of the study before we generated a stopping criterion.

Additional file 1: Tables S1a to S1d show the results of the two-stage stopping rules for the hypotheses, with a total sample size of 30 and 15 patients used in the first stage at *α* = 0.05 and 1 − *β* = 0.8 at a censoring rate *r*^*C*^ = 0.1. The corresponding errors at the early stage (the first stage) are *α*_1_ = 0.01 and *β*_1_ = 0.1 according to the error-spending-function method. In the first example (Additional file 1: Table S1a), a low RR of *p*_0_ = 0.05 and a short median TTE of *T*_0_ = 3 were set for the null hypothesis, and *p*_1_ = 0.2 and *T*_1_ = 4.5 were set for the alternative hypothesis. At the first stage with 15 patients, when the correlation between tumor response and the underlying true TTE is high (e.g., *ρ* = 0.8), the null hypothesis would be accepted and the treatment would be rejected if we observed: (1) no response with a median TTE of 6.9 or lower; (2) one response with a median TTE of 4.1 or lower; (3) two responses with a median TTE of 3.0 or lower; or (4) three responses with a median TTE of 2.7 or lower. For the final stage, the treatment would be considered efficacious if we observed: (1) a median TTE of 4.7 or higher with any number of responses; (2) one response with a median TTE of 4.6 or higher; (3) two or more responses with a median TTE of 4.5 or higher; (4) three or more responses with a median TTE of 4.1 or higher; (5) four or more responses with a median TTE of 3.2 or higher; or (6) five or more responses with any median TTE.

When implementing the proposed design for a specific trial, we suggest calculating the correlation based on RRs and TTEs from historical evidence. For example, if conducting a phase II trial for assessing bevacizumab activity, an angiogenesis inhibitor that slows the growth of new blood vessels, among patients with hepatocellular carcinoma (HCC), eight phase II trials on bevacizumab from 2006 to 2012 could be obtained using PubMed [22–29]. The results in Table 1 reveal that the Spearman correlation is estimated to be 0.90, which could be used as a correlation estimate for the proposed design. If historical results are unavailable, which could be the case for a relatively new drug, a search method could be applied to the interim data for estimating the most likely correlation. We first fixed the correlations and applied them in the copula setting to calculate the value of the likelihood function by using the interim data [30]. The correlation that provides the highest likelihood value would be an appropriate choice for the design. When interim data are unavailable at the design stage, the decision rules can be generated under several conditions (e.g., correlations from 0.1 to 0.9, in 0.1 increments) to provide a correlation range for reference. We may choose the maximal number of patients conservatively, and subsequently apply the likelihood function estimation to determine the most appropriate stopping rules after interim data are obtained. The calculation program for either historical results or interim data will be made available on our website http://www2.ccrb.cuhk.edu.hk.

**Table 1:** The tumor response and median PFS of phase II HCC trials on bevacizumab, 2006-2012

Article	Treatment arms	Median TTP/PFS (Month)	Response rate (%)
Zhu et al., 2006 [22]	GEMOX + Bevacizumab	5.3	20
Siegel et al., 2008 [23]	Bevacizumab	6.9	13.04
Thomas et al., 2009 [24]	Bevacizumab + Erlotinib	9.0	25
Hsu et al., 2010 [22]	Bevacizumab + Capecitabine	2.7	8.89
Sun et al., 2011 [26]	Bevacizumab + Capecitabine + Oxaliplatin	6.8	20
Kaseb et al., 2012 [27]	Bevacizumab + Erlotinib	7.2	23.73
Philip et al., 2012 [28]	Bevacizumab + Erlotinib	3.0	3.7
Yau et al., 2012 [29]	Bevacizumab + Erlotinib	1.5	0

## Results

### Simulation study

The simulation study was conducted to evaluate the operating characteristics of the proposed design. For each scenario, 1,000 samples were generated from the copula model (Appendix section A) to test the stopping criterion specified by P_0_, T_0_, P_1_, T_1_ and *ρ*. With a total of 30 patients and 15 in the early stage, the results in Table 2 (Rows 1–12) indicate that the proposed stopping rules can generally achieve the desired type I error and power when high RRs and a high hazard ratio between null and alternative are expected (P_0_ = 0.2, T_0_ = 4, P_1_ = 0.4 T_1_ = 8 and P_0_ = 0.3, T_0_ = 4, P_1_ = 0.5 T_1_ = 8). The type I error in low RR and low hazard ratio design (P_0_ = 0.05, T_0_ = 3, P_1_ = 0.2 T_1_ = 4.5) is higher than desired at the 5 % level and the power is slightly lower than 80 % when RR increased to P_0_ = 0.1, P_1_ = 0.3. When the sample size increased to n = 40 (the last eight rows in Table 2), the type I error and power improved to the acceptable level. In other words, when we expect a low tumor RR in the study (e.g., cytostatic trial), a sample size of 40 may be appropriate to achieve the desired type I error and power. If the expected RR is high (e.g., chemotherapy in multiple myeloma trials), 30 patients would be sufficient when using the proposed phase II design. The early stopping probability and expected sample size are also affected by a correlation between tumor response and TTE. The early stopping probability enhanced when correlation decreased, and thus less expected sample size was required with lower correlation between the two endpoints in the trial. This is reasonable because the more independent the two endpoints in the study are, the more information is available for statistical inference.

**Table 2:** Simulation results of two-stage design for testing H_0_: P ≤ P_0_ & T^*^_med_ ≤ T_0_ vs. H_1_: P > P_1_ or T^*^_med_ > T_1_ at the nominal level α = 0.05 and 1-β = 0.80^a^

Null		Alternative		Sample size^b^		Corr^c^				
P_0_	T_0_	P_1_	T_1_	n_1_	n	ρ	α	1-β	PET^d^	EN_0_^e^
0.05	3.0	0.20	4.5	15	30	0.8	0.065	0.801	0.786	18.210
						0.5	0.065	0.798	0.820	17.700
						0.2	0.069	0.801	0.853	17.205
						0.0	0.055	0.797	0.893	16.605
0.10	3.0	0.30	5.0	15	30	0.8	0.051	0.794	0.846	17.310
						0.5	0.052	0.790	0.884	16.740
						0.2	0.038	0.797	0.930	16.050
						0.0	0.026	0.791	0.949	15.765
0.20	4.0	0.40	8.0	15	30	0.8	0.046	0.818	0.861	17.085
						0.5	0.044	0.800	0.907	16.395
						0.2	0.033	0.807	0.945	15.825
						0.0	0.021	0.796	0.966	15.510
0.30	4.0	0.50	8.0	15	30	0.8	0.057	0.807	0.841	17.385
						0.5	0.037	0.795	0.911	16.355
						0.2	0.034	0.796	0.928	16.080
						0.0	0.028	0.802	0.950	15.750
0.05	3.0	0.20	4.5	20	40	0.8	0.049	0.807	0.880	22.400
						0.5	0.053	0.802	0.892	22.160
						0.2	0.044	0.797	0.916	21.680
						0.0	0.036	0.804	0.936	21.280
0.10	4.0	0.30	8.0	20	40	0.8	0.053	0.802	0.931	21.380
						0.5	0.044	0.806	0.930	21.400
						0.2	0.028	0.813	0.957	20.860
						0.0	0.025	0.805	0.969	20.620

^a^The censoring rates for time-to-event endpoint are set as 0.1 for both stopping rules and simulated data

^b^n_1_ and n denote the sample size in the first stage and the total sample size, respectively

^c^ρ denotes the correlation between response endpoint and time-to-event endpoint

^d^PET denotes the early stopping probability under H_0_

^e^EN_0_ denotes the expected sample size under H_0_

From the decision rules in Additional file 1: Tables S1a to S1d, a study agent could be declared to be active with a sufficiently high RR but with an extremely short median TTE, or if the median TTE is sufficiently long, but with a small number of responses. Another situation is if the treatment has a relatively high median TTE or RR but is claimed to be inactive, e.g., a trial with no response has a median TTE of 6.8 months at the first stage could be stopped early in the first block of Table S1a, despite 6.8 months being 2.3 months in excess of what is defined by the alternative hypothesis. We considered these clinically ambiguous situations in the simulation for assessing the performance of the generated decision rules. The overall type I (an inactive drug incorrectly claimed as active) and type II errors (an active drug incorrectly claimed as inactive) were still under the desired level (α <0.05, β <0.2), suggesting that the error of claiming active or inactive drugs with contradictory results is small.

In generating the decision rules, we employed the exponential distribution assumption for the TTE outcome. To assess the sensitivity of the distribution assumption for the TTE outcome, we performed another simulation where we assumed a more general Weibull distribution with a diverse shape parameter k. The Weibull distribution is equivalent to the exponential distribution when k = 1, implying a constant hazard during the study. The hazard decreases with time if k <1 and increases with time if k >1. The results in Table 3 indicate that the type I and type II errors increase if the decision rules based on the constant hazard assumption are applied, but they were observed to decrease. When the magnitude of hazard decrease is large (k = 0.5) and the total sample size is 40, the type I and type II errors deteriorate to unacceptable levels (α >0.1 and power <0.75). The type II error problem can be rectified by increasing the sample size in the severe hazard decrease situation (k = 0.5). However, this strategy has little effect on reducing type I error. When increased hazard is observed over time, the decision rules lead to fewer type I errors and power. In summary, only a decreased hazard situation will worsen the performance of the proposed design based on the exponential distribution assumption for TTE outcome.

**Table 3:** Sensitivity analysis with Weibull distribution assumed for TTE

Null		Alternative		Corr	Sample size		Shape parameter			
P_0_	T_0_	P_1_	T_1_	ρ	n_1_	n	K^a^	α	1-β	PET
0.05	3	0.2	4.5	0.8	20	40	1.0	0.048	0.808	0.891
							1.5	0.033	0.855	0.912
							1.2	0.036	0.823	0.901
							0.8	0.058	0.782	0.863
							0.5	0.128	0.719	0.794
0.05	3	0.2	4.5	0.8	30	60	1.0	0.030	0.817	0.958
							1.5	0.013	0.871	0.978
							1.2	0.028	0.846	0.959
							0.8	0.040	0.796	0.930
							0.5	0.115	0.782	0.825
0.05	3	0.2	4.5	0.8	40	80	1.0	0.016	0.847	0.977
							1.5	0.005	0.888	0.991
							1.2	0.008	0.863	0.990
							0.8	0.033	0.827	0.955
							0.5	0.102	0.818	0.855
0.3	4	0.5	8	0.8	20	40	1	0.047	0.817	0.884
							1.5	0.020	0.873	0.949
							1.2	0.035	0.834	0.912
							0.8	0.062	0.786	0.875
							0.5	0.121	0.710	0.793
0.3	4	0.5	8	0.8	30	60	1.0	0.020	0.837	0.965
							1.5	0.009	0.881	0.989
							1.2	0.014	0.851	0.983
							0.8	0.032	0.790	0.949
							0.5	0.090	0.742	0.849
0.3	4	0.5	8	0.8	40	80	1.0	0.020	0.857	0.975
							1.5	0.005	0.930	0.992
							1.2	0.008	0.881	0.990
							0.8	0.030	0.833	0.954
							0.5	0.096	0.803	0.852

^a^Shape parameter of Weibull distribution assumed for TTE: constant hazard if K = 1; increase hazard if K >1; decrease hazard if K <1

We also used the simulation study to compare the proposed design with the Zee et al. multinomial design, based on two binary endpoints, as well as with Simon’s optimal design, based on a single-tumor-response endpoint, with approximately the same sample size and hazard ratio of the event of interest, such as progression. For example, the scenario with an early progression rate of 0.6 in H_0_ and of 0.4 in H_1_ was considered to have a hazard ratio of 1.5 in the progression, corresponding to the situation of T_0_ = 3 and T_1_ = 4.5 in Table 2. The results in Table 4 indicate that the probability of stopping the study early is similar to the multinomial design and Simon’s optimal design when tumor RRs for null and alternative hypotheses are P_0_ = 0.1 versus P_1_ = 0.3. With the lower (P_0_ = 0.05 and P_1_ = 0.2) or higher tumor RR (P_0_ = 0.3 and P_1_ = 0.5), the proposed design showed higher early stopping probability compared with both the multinomial design and Simon’s optimal design.

**Table 4:** Comparison with Simon’s optimal design and Zee’s multinomial design

P_0_	T_0_	P_1_	T_1_	*ρ* ^a^	n_1_	n	Type^b^	PET	α	1-β	EN_0_
0.05	3	0.2	4.5	0.2	21	41	Proposed design	0.855	0.055	0.903	23.900
							Simon	0.720	0.046	0.902	26.700
							Multinomial	0.770	0.072	0.934	24.600
0.1	3	0.3	5	0.5	10	29	Proposed design	0.788	0.052	0.792	14.028
							Simon	0.740	0.047	0.805	15.000
							Multinomial	0.655	0.039	0.792	16.900
0.2	4	0.4	8	0.5	13	43	Proposed design	0.874	0.053	0.811	16.780
							Simon	0.750	0.049	0.800	20.600
							Multinomial	0.723	0.049	0.866	21.300
0.3	4	0.5	8	0.5	15	46	Proposed design	0.880	0.042	0.808	18.720
							Simon	0.720	0.049	0.803	23.600
							Multinomial	0.763	0.062	0.848	22.100

^a^Correlation parameter is set to approximate Zee’s mulatinomial design

^b^Sample size in multinomial design is slightly different (Zee et al., 1999)

^c^PET and EN0 are obtained from Table III in Whitehead (2014)

We also compared the proposed design with those based on a single TTE endpoint [11, 12]. For example, we compared a null hypothesis with an RR of 0.1 and a median TTE of 3 versus an alternative hypothesis with an RR of 0.3 and a median TTE of 5, using 34 patients in the first stage and 68 patients in the final stage. The expected sample size of the approximate survival method [12] using the TTE alone was 51, with a type I error equal to 0.097 and a power of 0.850, whereas our proposed method with mixed RR and TTE endpoints yields a smaller expected sample size of 34.6, a type I error of 0.017, and a power of 0.881. For the same hypothesis using 52 patients in the first stage and 81 patients in the final stage, the expected sample size based on the TTE endpoint alone [11] was 63.5 with a type I error of 0.122 and a power of 0.935, and our proposed method based on mixed RR and TTE endpoints yields a smaller expected sample size of 52.6 in the presence of a type I error of 0.019 and a power of 0.979. The results showed that the mixed-endpoints design has a smaller expected sample size and higher early stopping probability according to H_0_, indicating that the proposed design is more likely to stop an inactive agent than those based on a single TTE endpoint.

### Discussion of application

Numerous multistage designs have recently been developed for phase II clinical trials. However, these designs are based either on a tumor-response endpoint alone or a TTE endpoint alone (e.g., progression-free survival). This, however, may not be the optimal strategy for evaluating the efficacy of study treatments because a natural correlation could exist between tumor response and the TTE [20], and because abandoning either endpoint may cause severe information loss. Although Zee et al. [16] and Sun et al. [17] proposed multinomial designs to incorporate tumor response and EPD information, the dichotomous EPD endpoint does not permit fully extracting the information from a study, compared with the TTE endpoint. We generalized these designs to integrate the tumor response endpoint and the TTE endpoint, to fully use the information in the study and to generate efficient stopping rules. We assumed a Gaussian copula to describe the dependent structure between binary tumor response and continuous TTE, which is similar to the binomial-exponential setting used by de Leon and Wu [30]. Because no analytic solution exists, we employed a simulation-based method to generate the stopping rules for tumor response and median TTE under various fixed correlations between endpoints. The results of a two-stage design showed that the correlation has an effect on the stopping boundary at the final stage and on the decision criterion for early stopping in the first stage. As the correlation decreases, the boundary for early stopping also decreases, meaning that a shorter median TTE or smaller RR is required to stop the study at the interim analysis. When we apply the proposed design in practice, the correlation between RR and TTE can be estimated from the results of previous studies. When historical data are unavailable, particularly for a new drug, the preliminary study data can be used to estimate the correlation based on the copula likelihood function of the interim data. To make this method more applicable, we developed an R program for calculating the correlation.

The interim assessment can be planned at the time when half of the patients achieve progression, or the time corresponding to each response in the early stopping rules, whichever is shorter. For example, if H_0_: P ≤0.05 and TTP^*^_med_ ≤3 versus H_1_: P >0.2 or TTP^*^_med_ >4.5 and correlation ρ is set as 0.2 to approximate the correlation in the Zee multinomial design, the interim assessments can be conducted at the time when half of the patients develop progression or 4.5 months, whichever is shorter, when one response is observed. Compared with the Zee multinomial design, one advantage of the proposed design is that the interim assessment does not rely on the progression time and hence we would be able to decide earlier, which is particularly useful for trials with long time to progression (e.g., prostate cancer trials). Compared with those designs based on a single TTE, the proposed design has the ability to reduce the potential long waiting time for assessing the TTE outcome if response increases.

Similar to the two-stage design based on the TTE endpoint, the proposed design also has the limitation of requiring a longer wait period than tumor response to obtain the estimated median TTE [14]. However, the proposed design with the tumor-response endpoint could mitigate this limitation to a certain extent. For example, if a phase II trial is conducted using the sample size and hypotheses in the last block of Table S1a (correlation = 0), the clinician must wait 6.6 months or until the occurrence of eight events (median of 15 patients), whichever is shorter, when no response is observed at the first stage. If the eighth event occurs before 6.6 months without response, the trial can be stopped for futility; if less than eight events occur at 6.6 months, the study can be continued to stage II. In the event of one response the waiting period can be reduced to 4.9 months or until eight events have been observed. If the required period for the TTE endpoint evaluation is not excessively long in some advanced cancer studies, the proposed design has the potential to accelerate the inference, which may improve the efficiency of phase II clinical trials. Therefore, the introduction of tumor response in the proposed design could reduce the potential waiting time compared with designs based on a single TTE endpoint.

In our proposed design, we only allow early stopping for futility, because investigators commonly choose to continue the study in practice, even if early rejection criteria of the null hypothesis are fulfilled. Considering that the study would not be stopped early when either of the endpoints is extremely promising, we used the early rejection rules to adaptively bind the early acceptance rules to obtain reasonable stopping rules for futility. With 30 patients, the simulation results indicate that the proposed stopping rules can generally achieve the desired type I error of 5 % and power of 80 % when high RRs and a high hazard ratio between null and alternative hypotheses are expected. The type I error in the low RR and low hazard ratio design is higher than the desired 5 % level. If the sample size increases to 40, the desired type I and type II errors can be maintained in various situations. This implies that a sample size of 30 could be sufficient to achieve the desired type I and type II error levels for trials with a high expected RR and a high hazard ratio; if a low RR and low hazard ratio are expected, a slightly larger sample size of 40 may be adequate. If early stopping for activity is allowed, the boundary of early rejection may overlap with the boundary of early acceptance. This means that the conclusion of the effectiveness of the study treatment may be contradictory as being both efficacious and inactive in the first stage, because of the flexibility (or characteristics) of two diverse quadrants of parameter space in the response and the hazard of the TTE. In this case, the adaptive approach based on early rejection rules may be useful for eliminating the overlap.

The proposed design assumes that both the response endpoint and the TTE endpoint indicate study agent activity, which may be true in a targeted drug setting with unknown clinical activity. In the case of a cytostatic drug, possible tumor shrinkage or response may still indicate drug activity, although inhibited tumor growth is primarily targeted and the TTE endpoint is commonly adopted in the assessment. Thus, the null hypothesis can be rejected and the drug can be accepted if either of these endpoints exceeds the required level, despite another endpoint indicating futility. Tumor response is typically positively correlated with the TTE, and an extremely high RR (e.g., >60 %) coinciding with an extremely small median TTE (e.g., one month) is unlikely. In the event of this extreme situation occurring, further investigation of the study agent should be conducted to determine the mechanism behind the unexpected observation.

Although exponential distribution is commonly assumed for patient survival [31], the constant hazard implied by exponential distribution may be incorrect in practical trials. The sensitivity analysis indicated that the proposed design based on the exponential assumption for TTE is applicable in practical phase II cancer trials where the hazard increases with time. However, if the decreased hazard is observed in the study, the chance of incorrectly concluding that an inactive drug is active (type I error) or an active drug is inactive (type II error) may exceed the expected level. Furthermore, the assessment period, typically scheduled cyclically, may also affect the estimate of the median TTE [32]. The Panageas’s [32] simulation results showed that the commonly used upper-limit progression time (where the progression date is defined as the date at which progression is first detected during the assessment cycle) could overestimate the true median TTP or PFS, thus affecting the statistical inference only at the final stage rather than at the early stage. This is because only early acceptance of null hypotheses is allowed in the proposed design, and the true median TTE, which is shorter than the estimated TTE, still fulfills the early stopping criteria. Following the recommendation in the Panageas’s study [32], the upper limit and lower limit (where the progression date is defined as the date before one cycle at which progression is first observed) can be combined to draw the conclusion. The lower limit of the TTE can be employed to validate the inference based on the commonly used upper limit of the TTE, when observed results suggest rejecting the null hypothesis.

We also compared the early stopping probability and the expected sample size of our design with the Zee et al. multinomial design based on tumor response and the binary progression event, as well as with Simon’s optimal design based on single tumor response. The simulation results showed that the probability of stopping a study early is consistently higher, yielding a smaller expected sample size than Simon’s design, thereby indicating that integrating the tumor response endpoint and the TTE endpoint yields more efficient stopping rules than a design that has only a single tumor-response endpoint. When the expected RR is P_0_ = 0.1 versus P_1_ = 0.3, the performance of the proposed design is approximately equal to the Zee multinomial design, in early stopping probability and expected sample size. Unlike in other scenarios where P_0_ = 0.05, P_1_ = 0.2 and P_0_ = 0.3, P_1_ = 0.5, our design has a higher early stopping probability and a smaller expected sample size. Therefore, incorporating TTE into the design exhibits superior performance compared with the Zee design using binary endpoints in the expected sample size. Furthermore, compared with two-stage single arm designs based on a single TTE endpoint [11, 12], the simulation results also indicated the superiority of the proposed design in expected sample size and early stopping probability according to the null hypothesis.

## Conclusions

The proposed single-arm phase II design extends the Zee multinomial design to fully use the information for various types of endpoint, where the TTE endpoint could be progression-free survival. The advantage of this design is its applicability either to cytotoxic or noncytotoxic treatment studies when the median TTE can be measured in the trials. Our proposed design requires a smaller expected sample size than other methods for maintaining the desired statistical properties. Therefore, when a single-arm design is adopted in a phase II trial setting, which may be due to limited patient availability or studies investigating a therapy with only a single agent [33], it would be a superior choice for drug screening in phase II clinical trials.

## Additional file

Additional file 1:
**Table S1.**a Two-stage stopping rules for response and time-to-event endpoints^a^ with H_0_: P ≤ 0.05 and T^*^_med_ ≤3 vs. H_1_: P >0.2 or T^*^_med_ >4.5 (α = 0.05, 1-β = 0.80). **Table S1.**b Two-stage stopping rules for response and time-to-event endpoints with H_0_: P ≤0.1 and T^*^_med_ ≤3 vs. H_1_: P >0.3 or T^*^_med_ >5 (α = 0.05, 1-β = 0.80). Table S1c Two-stage stopping rules for response and time-to-event endpoints with H_0_: P ≤0.2 and T^*^_med_ ≤4 vs. H_1_: P >0.4 or T^*^_med_ >8 (α = 0.05, 1-β = 0.80). Table S1d Two-stage stopping rules for response and time-to-event endpoints with H_0_: P ≤0.3 and T^*^_med_ ≤4 vs. H_1_: P >0.5 or T^*^_med_ >8 (α = 0.05, 1-β = 0.80).

## Appendix

### A. Copula model

Suppose that there are N patients accrued in the study, for the *i*^th^ patient we denote the observed binary tumor response outcome by *Y*_*i*_ with value 1 for responders and value 0 for non-responders, and the underlying true time-to-event by T_i_^*^ which is assumed to follow an exponential distribution exp (λ). Furthermore, the binary tumor response is determined by a latent normal variable *X*_*i*_^*^ through the probit model

A1 $$ {Y}_i=\left\{\begin{array}{l}\begin{array}{cc}\hfill 0,\hfill & \hfill \mathrm{if}\ {X}_i^{*}\in \left(-\infty,\ \gamma \right)\hfill \end{array}\\ {}\begin{array}{cc}\hfill 1,\hfill & \hfill \mathrm{if}\ {X}_i^{*}\in \left[\gamma, + \infty \right)\hfill \end{array}\end{array}\right. $$

where *γ* is the unknown threshold that could be determined by the pre-specified RR in the hypothesis setting. For example, if the RECIST [34] response rates to axitinib in sorafenib-refractory metastatic renal cell carcinoma are set as p_0_ = 0.08 and p_1_ = 0.2 in Rini et al. [35], then *γ* will be 1.41 and 0.84 for null and alternative hypotheses, respectively. For the unobservable time-to-event T_i_^*^, we define the censoring time T_i_^C^ such that T_i_^*^ could be observed only if T_i_^*^ ≤ T_i_^C^, that is we observe *T*_*i*_ = min{T_i_^*^, T_i_^C^}. Considering the categorical nature of the tumor response endpoint, we follow de Leon and Wu’s [30] copula method to model the dependence between these two endpoints by assuming the correlation between the underlying normal variable *X*_*i*_^*^ and the true time-to-event *T*_*i*_^*^. One advantage of copula is that it allows one to easily model the marginal distribution of random vectors and their correlation separately. Specifically in our design, the Gaussian copula is used to describe the dependence between *X*_*i*_^*^ and T_i_^*^ once the marginal distributions are assumed, such that the joint distribution is

A2 $$ {F}_{X_i^{*},{T}_i^{*}}\left(x,t\right)={\varPhi}_2\left({\varPhi}^{-1}\left\{\varPhi (x)\right\},{\varPhi}^{-1}\left\{{F}_{T_i^{*}}(t)\right\};\rho \right) $$

where *Φ* is the standard normal distribution representing the marginal distribution of *X*_*i*_^*^, *Φ*_2_ is the standard bivariate normal distribution with correlation *ρ*, and ${F}_{T_i^{*}}(t)=1-{e}^{-\lambda t}$ is the marginal distribution of true time-to-event T_i_^*^ with hazard *λ*. The correlation *ρ* describes the dependence between response and the TTE outcome, which is analogous to the polyserial correlation defined by Drasgow [36]. Under the Gaussian copula, the joint distribution of the observed tumor response outcome *Y*_*i*_ and the true time-to-event T_i_^*^ is

A3 $$ P\left({Y}_i=y,{T}_i^{*}\le t\right)=\left\{\begin{array}{l}\begin{array}{cc}\hfill {F}_{X_i^{*},{T}_i^{*}}\left(\gamma, t\right)\ \hfill & \hfill \begin{array}{cc}\hfill \hfill & \hfill \begin{array}{cc}\hfill \begin{array}{ccc}\hfill \hfill & \hfill \hfill & \hfill \hfill \end{array}\hfill & \hfill \begin{array}{cc}\hfill \hfill & \hfill \mathrm{if}\ y=0\hfill \end{array}\hfill \end{array}\hfill \end{array}\hfill \end{array}\ \\ {}{F}_{T_i^{*}}(t)-{F}_{X_i^{*},{T}_i^{*}}\left(\gamma, t\right)\begin{array}{ccc}\hfill \begin{array}{cc}\hfill \hfill & \hfill \hfill \end{array}\hfill & \hfill \hfill & \hfill \kern0.75em \mathrm{if}\ y=1\hfill \end{array}\end{array}\right. $$

where ${F}_{X_i^{*},{T}_i^{*}}$ is defined in (A2).

### B. Stopping rule generation procedure for multi-stage design

For a K-stage design, suppose one decides to accrue n_i_ patients in the *i*th stage such that the total number of patients *N* = n_1_ + n_2_ + … + n_K_. Let *s*_*i*_, *i* = 1, …, *K*, denote the number of patients with response in stage i; and *t*_*i*_^*med*^, *i* = 1, …, *K*, denote the Kaplan-Meier median based on all ${N}_i = {\displaystyle \sum_{j=1}^i}{n}_i$ observed time-to-event up to the *i*th stage. Furthermore, we denote the set of acceptance criteria of the null hypothesis for response at each stage by (*a*_1_^*Y*^, *a*_2_^*Y*^,…, *a*_*K*_^*Y*^) and for time-to-event by (*a*_1_^*T*^, *a*_2_^*T*^,…, *a*_*K*_^*T*^). Because the early acceptance of study agent is usually not allowed in practical trials, the decision rules for rejecting null hypotheses are only applied at the final stage. Denote the set of rejection criteria of the null hypothesis for response by *r*^*Y*^ and the rejection criteria for time-to-event by *r*^*T*^, a general multi-stage testing procedure is defined as follows. In stage g (g = 1, 2,…, K):

Accept *H*_0_ and conclude that the study treatment is not efficacious if

$$ {\displaystyle \sum_{i=1}^g{s}_i}\le {a}_g^Y $$ and *t*_*g*_^*med*^ ≤ *a*_*g*_^*T*^

Continue to accrue another n_g+1_ patient for (*g* + 1)^th^ stage trial if

$$ {\displaystyle \sum_{i=1}^g}{s}_i\ge {a}_g^Y $$ or *t*^*med*^ ≥ *a*_*g*_^*T*^

At the final stage, reject *H*_0_ and conclude that the study treatment is efficacious if

$$ {\displaystyle \sum_{i=1}^g}{s}_i\ \ge {r}^Y $$ or *t*^*med*^ ≥ *r*^*T*^

At the *g*th stage, the Type I error (*α*_*g*_) and the Type II error (*β*_*g*_) are adjusted by using the error spending function in Lan and DeMets [37] and Zee et al. [16] so that the overall error rates can be controlled at a pre-specified level. Specifically, ${\alpha}_g=\left[1-\varPhi \left({Z}_{1-\alpha }/\sqrt{N_g/N}\right)\right]$ and ${\beta}_g=\left[1-\varPhi \left({Z}_{1-\beta }/\sqrt{N_g/N}\right)\right]$, where *Z*_1 − *α*_ and *Z*_1 − *β*_ are the (1- α)% quantile and (1- β)% quantile of standard normal distribution, respectively, and *N*_*g*_ = n_1_ + n_2_ + … + n_g_. To determine the *r*^*Y*^ , *r*^*T*^, *a*_*g*_^*Y*^, *a*_*g*_^*T*^, for given *α*_*g*_, *β*_*g*_ and corresponding *p*_0_, *p*_1_, *T*_0_, *T*_1_ in the hypothesis setting, the simulation-based approach is employed:

Step 1. Generate 10,000 samples from the copula (A3) under the null hypothesis, with a fixed size for the *g*th stage; calculate the corresponding statistic ${\displaystyle \sum_{i=1}^g{s}_i}$, *t*_*g*_^*med*^ for each of the samples.

Step 2. Compile the table of empirical distribution $P\left({\displaystyle \sum_i^g{Y}_i}\ge k,{T}_g^{med}\ge t\right)$ by using one integer increment for *k* and 0.1 unit increment for *t*.

Step 3. Compile the table of empirical distribution $P\left({\displaystyle \sum_i^g{Y}_i}\le k,{T}_g^{med}\le t\right)$ by using the same increment grid as in Step 2.

Step 4. Use the copula (A3) to generate another 10,000 samples under the alternative hypothesis for stage g.

Step 5. Compile the same tables of empirical distribution $P\left({\displaystyle \sum_i^g{Y}_i}\ge k,{T}_g^{med}\ge t\right)$ and $P\left({\displaystyle \sum_i^g{Y}_i}\le k,{T}_g^{med}\le t\right)$ based on 10,000 samples under the alternative hypothesis.

Step 6. Determine the decision criterion for the *g*^th^ stage based on the distribution tables in step 5. With the spent error *α*_*g*_ and *β*_*g*_, we search the distribution table in step 5 to find the decision boundary values *a*_*g*_^*Y*^, *a*_*g*_^*T*^ for accepting null hypotheses such that $\mathrm{P}\left({\displaystyle \sum_i^g}{Y}_i\le,\ {T}_g^{med}\le \right)\kern0.37em \le {\beta}_g$ and $\mathrm{P}\left({\displaystyle \sum_i^g}{Y}_i\le +1,\ {T}_g^{med}\le +0.1\right)\kern0.37em >{\beta}_g$. At the last stage K, the distribution tables in step 2 and step 3 are searched to find the decision boundary values *r*^*Y*^, *r*^*T*^ for rejecting null hypotheses such that $\mathrm{P}\left({\displaystyle \sum_i^K}{Y}_i\ge {r}^Y,\ {T}_K^{med}\ge {r}^T\ \right)\kern0.37em \le \upalpha$ and $\mathrm{P}\left({\displaystyle \sum_i^K}{Y}_i\ge {r}^Y-1,\ {T}_K^{med}\ge {r}^T-0.1\right)\kern0.37em >\upalpha$.

Because two different quadrants are left in the parameter space of response probability and hazard of TTE, impractical stopping rules may occur as a result, i.e., design may asks for early termination for futility when there is zero response with a long median survival; or a very short median survival with high responses. However, the drug/treatment is unlikely to be concluded as inactive if either of the two endpoints shows promising value (i.e., either high RR or long median TTE) in practice. To overcome this problem, the stopping rules for activity, which are generated in the same way as those in the last stage but with spent error α_*g*_, are proposed to be adaptively bounded by the rules for futility, so that the cut-off values for early acceptance of the null hypothesis cannot exceed those for early rejection. For example, the early acceptance rules of the null hypothesis could be 1 or less response with any median TTE, and thus the trial with zero response and 7.5 month median TTE at first stage will be stopped early due to futility. But a long median TTE could have been inferred as active if the early stopping rule for activity is median TTE of 6.0 or longer with any response, indicating that the 7.5 month median TTE leads us to study the treatment further rather than stopping early, even though the number of responses is below the futility boundary. Therefore, the TTE “tail” of the final early acceptance boundary is cut off at 1 or fewer response with 6.0 or shorter median TTE, to avoid the “dilemma” of being concluded as both active and inefficacious. This adaption is also applied to the response “tail” of the early stopping boundary.

## Abbreviations

EPD: Early progressive disease
OS: Overall survival
*P*: Response rate
PFS: Progression-free survival
r: Correlation coefficient
*r*^*c,*^: Censoring rate
RECIST: Response evaluation criteria in solid tumors
RR: Response rate
*T*_*0*_*,*: Median time for the null hypothesis
*T*_*1*_: Median time for the alternative hypothesis
TTE: Time-to-event
TTP: Time-to-progression
*T**_*med*_: Median time-to-event
*T*^*C*^: Censoring time
*λ*: Hazard
*p*_*0*_*,*: Response rate for the null hypothesis
*p*_*1*_*,*: Response rate for the alternative hypothesis
α: Type I error
β: Type II error
