# Supplementary material for: Mixed response and time-to-event endpoints for multistage single-arm phase II design
Source: Trials. 2015 Jun 4;16:250. doi: 10.1186/s13063-015-0743-9 (PMC4460691; doi:10.1186/s13063-015-0743-9)
Supplement: Additional file 1: — Table S1.a Two-stage stopping rules for response and time-to-event endpointsa with H0: P ≤ 0.05 and T* med ≤3 vs. H1: P >0.2 or T* med >4.5 (α = 0.05, 1-β = 0.80). Table S1.b Two-stage stopping rules for response and time-to-event endpoints with H0: P ≤0.1 and T* med ≤3 vs. H1: P >0.3 or T* med >5 (α = 0.05, 1-β = 0.80). Table S1c Two-stage stopping rules for response and time-to-event endpoints with H0: P ≤0.2 and T* med ≤4 vs. H1: P >0.4 or T* med >8 (α = 0.05, 1-β = 0.80). Table S1d Two-stage stopping rules for response and time-to-event endpoints with H0: P ≤0.3 and T* med ≤4 vs. H1: P >0.5 or T* med >8 (α = 0.05, 1-β = 0.80). [file 13063_2015_743_MOESM1_ESM.docx]

Table 1a. Two-stage Stopping rules for response and time-to-event endpoints^a^ with H_0_: P ≤ 0.05 & T^*^_med_ ≤ 3 vs. H_1_: P >0.2 or T^*^_med_ >4.5 (α=0.05, 1-β=0.80).

|  | Corr^b^ |  | Stage I^c^ | | | |  | Stage II | |  |
| --- | --- | --- | --- | --- | --- | --- | --- | --- | --- | --- |
|  | $\rho$ |  |  | <=a_11_/n_1_ | <=T_a_ |  |  | >=r_21_/n | >=T_r_ |  |
|  | 0.8 |  |  | 0/15 | 6.9 |  |  | - | 4.7 |  |
|  |  |  |  | 1/15 | 4.1 |  |  | 1/30 | 4.6 |  |
|  |  |  |  | 2/15 | 3.0 |  |  | 2/30 | 4.5 |  |
|  |  |  |  | 3/15 | 2.7 |  |  | 3/30 | 4.1 |  |
|  |  |  |  |  |  |  |  | 4/30 | 3.2 |  |
|  |  |  |  |  |  |  |  | 5/30 | - |  |
|  | 0.5 |  |  | 0/15 | 6.8 |  |  | - | 4.7 |  |
|  |  |  |  | 1/15 | 4.3 |  |  | 1/30 | 4.6 |  |
|  |  |  |  | 2/15 | 3.2 |  |  | 2/30 | 4.4 |  |
|  |  |  |  | 3/15 | 2.8 |  |  | 3/30 | 4.0 |  |
|  |  |  |  |  |  |  |  | 4/30 | 3.0 |  |
|  |  |  |  |  |  |  |  | 5/30 | - |  |
|  | 0.2 |  |  | 0/15 | 6.7 |  |  | - | 4.7 |  |
|  |  |  |  | 1/15 | 4.5 |  |  | 1/30 | 4.6 |  |
|  |  |  |  | 2/15 | 3.3 |  |  | 2/30 | 4.3 |  |
|  |  |  |  | 3/15 | 2.9 |  |  | 3/30 | 3.8 |  |
|  |  |  |  |  |  |  |  | 4/30 | 2.7 |  |
|  |  |  |  |  |  |  |  | 5/30 | - |  |
|  | 0 |  |  | 0/15 | 6.6 |  |  | - | 4.7 |  |
|  |  |  |  | 1/15 | 4.9 |  |  | 1/30 | 4.6 |  |
|  |  |  |  | 2/15 | 3.4 |  |  | 2/30 | 4.3 |  |
|  |  |  |  | 3/15 | 3.0 |  |  | 3/30 | 3.7 |  |
|  |  |  |  |  |  |  |  | 4/30 | 2.8 |  |
|  |  |  |  |  |  |  |  | 5/30 | - |  |

^a^ Censoring rates for time-to-event endpoint are set as 0.1 under both H_0_ and H_1_.

^b^ Corr denotes the correlation between response endpoint and time to event endpoint.

^c^ a_11_ and r_21_ denote the acceptance boundary for response at 1^st^ stage and the rejection boundary of H_0_ at *2^nd^* stage; T_a_ and T_r_

denote the acceptance boundary and rejection boundary of H_0_ for median time-to-event; n_1_ denotes the sample

size in the 1^st^ stage and n denotes the total sample size.

Table 1b. Two-stage Stopping rules for response and time-to-event endpoints with H_0_: P ≤ 0.1 & T^*^_med_ ≤3 vs. H_1_: P >0.3 or T^*^_med_ >5 (α=0.05, 1-β=0.80).

|  | Corr |  | Stage I | | | |  | Stage II | |  |
| --- | --- | --- | --- | --- | --- | --- | --- | --- | --- | --- |
|  | $\rho$ |  |  | <=a_11_/n_1_ | <=T_a_ |  |  | >=r_21_/n | >=T_r_ |  |
|  | 0.8 |  |  | 1/15 | 6.8 |  |  | - | 4.7 |  |
|  |  |  |  | 2/15 | 4.8 |  |  | 3/30 | 4.6 |  |
|  |  |  |  | 3/15 | 3.5 |  |  | 4/30 | 4.5 |  |
|  |  |  |  | 4/15 | 3.1 |  |  | 5/30 | 4.2 |  |
|  |  |  |  | 5/15 | 3.0 |  |  | 6/30 | 3.6 |  |
|  |  |  |  |  |  |  |  | 7/30 | - |  |
|  | 0.5 |  |  | 1/15 | 6.8 |  |  | - | 4.7 |  |
|  |  |  |  | 2/15 | 5.4 |  |  | 3/30 | 4.6 |  |
|  |  |  |  | 3/15 | 3.8 |  |  | 4/30 | 4.4 |  |
|  |  |  |  | 4/15 | 3.3 |  |  | 5/30 | 4.0 |  |
|  |  |  |  | 5/15 | 3.1 |  |  | 6/30 | 3.3 |  |
|  |  |  |  |  |  |  |  | 7/30 | - |  |
|  | 0.2 |  |  | 1/15 | 6.7 |  |  | - | 4.7 |  |
|  |  |  |  | 2/15 | 6.1 |  |  | 2/30 | 4.6 |  |
|  |  |  |  | 3/15 | 4.0 |  |  | 3/30 | 4.5 |  |
|  |  |  |  | 4/15 | 3.4 |  |  | 4/30 | 4.2 |  |
|  |  |  |  | 5/15 | 3.1 |  |  | 5/30 | 3.8 |  |
|  |  |  |  |  |  |  |  | 6/30 | 3.1 |  |
|  |  |  |  |  |  |  |  | 7/30 | - |  |
|  | 0 |  |  | 1/15 | 6.8 |  |  | - | 4.7 |  |
|  |  |  |  | 2/15 | 6.0 |  |  | 2/30 | 4.6 |  |
|  |  |  |  | 3/15 | 4.3 |  |  | 3/30 | 4.4 |  |
|  |  |  |  | 4/15 | 3.6 |  |  | 4/30 | 4.1 |  |
|  |  |  |  | 5/15 | 3.3 |  |  | 5/30 | 3.7 |  |
|  |  |  |  |  |  |  |  | 6/30 | 2.9 |  |
|  |  |  |  |  |  |  |  | 7/30 | - |  |
|  |  |  |  |  |  |  |  |  |  |  |

Table 1c. Two-stage Stopping rules for response and time-to-event endpoints with H_0_: P ≤ 0.2 & T^*^_med_ ≤ 4 vs. H_1_: P > 0.4 or T^*^_med_ > 8 (α=0.05, 1-β=0.80).

|  | Corr |  | Stage I | | | |  | Stage II | |  |
| --- | --- | --- | --- | --- | --- | --- | --- | --- | --- | --- |
|  | $\rho$ |  |  | <=a_11_/n_1_ | <=T_a_ |  |  | >=r_21_/n | >=T_r_ |  |
|  | 0.8 |  |  | 3/15 | 8.8 |  |  | - | 6.3 |  |
|  |  |  |  | 4/15 | 5.8 |  |  | 7/30 | 6.2 |  |
|  |  |  |  | 5/15 | 5.1 |  |  | 8/30 | 6.0 |  |
|  |  |  |  | 6/15 | 4.9 |  |  | 9/30 | 5.6 |  |
|  |  |  |  | 7/15 | 4.8 |  |  | 10/30 | 5.0 |  |
|  |  |  |  |  |  |  |  | 11/30 | - |  |
|  | 0.5 |  |  | 3/15 | 9.0 |  |  | - | 6.2 |  |
|  |  |  |  | 4/15 | 6.7 |  |  | 5/30 | 6.1 |  |
|  |  |  |  | 5/15 | 5.5 |  |  | 6/30 | 6.0 |  |
|  |  |  |  | 6/15 | 5.1 |  |  | 7/30 | 5.8 |  |
|  |  |  |  | 7/15 | 4.9 |  |  | 8/30 | 5.6 |  |
|  |  |  |  |  |  |  |  | 9/30 | 5.1 |  |
|  |  |  |  |  |  |  |  | 10/30 | 4.3 |  |
|  |  |  |  |  |  |  |  | 11/30 | - |  |
|  | 0.2 |  |  | 3/15 | 9.0 |  |  |  | 6.3 |  |
|  |  |  |  | 4/15 | 7.4 |  |  | 5/30 | 6.2 |  |
|  |  |  |  | 5/15 | 5.9 |  |  | 6/30 | 6.0 |  |
|  |  |  |  | 6/15 | 5.3 |  |  | 7/30 | 5.8 |  |
|  |  |  |  | 7/15 | 5.0 |  |  | 8/30 | 5.4 |  |
|  |  |  |  |  |  |  |  | 9/30 | 4.8 |  |
|  |  |  |  |  |  |  |  | 10/30 | 3.9 |  |
|  |  |  |  |  |  |  |  | 11/30 | - |  |
|  | 0 |  |  | 3/15 | 9.1 |  |  | - | 6.3 |  |
|  |  |  |  | 4/15 | 7.8 |  |  | 3/30 | 6.2 |  |
|  |  |  |  | 5/15 | 6.3 |  |  | 5/30 | 6.1 |  |
|  |  |  |  | 6/15 | 5.4 |  |  | 6/30 | 5.9 |  |
|  |  |  |  | 7/15 | 5.1 |  |  | 7/30 | 5.5 |  |
|  |  |  |  |  |  |  |  | 8/30 | 5.2 |  |
|  |  |  |  |  |  |  |  | 9/30 | 4.6 |  |
|  |  |  |  |  |  |  |  | 10/30 | 3.2 |  |
|  |  |  |  |  |  |  |  | 11/30 | - |  |
|  |  |  |  |  |  |  |  |  |  |  |

Table 1d. Two-stage Stopping rules for response and time-to-event endpoints with H_0_: P ≤ 0.3 & T^*^_med_ ≤4 vs. H_1_: P > 0.5 or T^*^_med_ > 8 (α=0.05, 1-β=0.80).

|  | Corr |  | Stage I | | | |  | Stage II | |  |
| --- | --- | --- | --- | --- | --- | --- | --- | --- | --- | --- |
|  | $\rho$ |  |  | <=a_11_/n_1_ | <=T_a_ |  |  | >=r_21_/n | >=T_r_ |  |
|  | 0.8 |  |  | 4/15 | 9.0 |  |  | - | 6.3 |  |
|  |  |  |  | 5/15 | 6.6 |  |  | 10/30 | 6.2 |  |
|  |  |  |  | 6/15 | 5.3 |  |  | 11/30 | 6.0 |  |
|  |  |  |  | 7/15 | 4.9 |  |  | 12/30 | 5.8 |  |
|  |  |  |  | 9/15 | 4.8 |  |  | 13/30 | 5.2 |  |
|  |  |  |  |  |  |  |  | 14/30 | - |  |
|  | 0.5 |  |  | 4/15 | 9.2 |  |  | - | 6.3 |  |
|  |  |  |  | 5/15 | 7.7 |  |  | 6/30 | 6.2 |  |
|  |  |  |  | 6/15 | 5.8 |  |  | 8/30 | 6.2 |  |
|  |  |  |  | 7/15 | 5.2 |  |  | 9/30 | 6.1 |  |
|  |  |  |  | 8/15 | 4.9 |  |  | 10/30 | 6.0 |  |
|  |  |  |  | 9/15 | 4.8 |  |  | 11/30 | 5.8 |  |
|  |  |  |  |  |  |  |  | 12/30 | 5.4 |  |
|  |  |  |  |  |  |  |  | 13/30 | 4.8 |  |
|  |  |  |  |  |  |  |  | 14/30 | - |  |
|  | 0.2 |  |  | 4/15 | 9.1 |  |  | - | 6.3 |  |
|  |  |  |  | 5/15 | 8.3 |  |  | 5/30 | 6.2 |  |
|  |  |  |  | 6/15 | 6.4 |  |  | 7/30 | 6.2 |  |
|  |  |  |  | 7/15 | 5.5 |  |  | 8/30 | 6.0 |  |
|  |  |  |  | 8/15 | 5.2 |  |  | 9/30 | 5.9 |  |
|  |  |  |  | 9/15 | 4.9 |  |  | 10/30 | 5.7 |  |
|  |  |  |  |  |  |  |  | 11/30 | 5.4 |  |
|  |  |  |  |  |  |  |  | 12/30 | 5.0 |  |
|  |  |  |  |  |  |  |  | 13/30 | 4.4 |  |
|  |  |  |  |  |  |  |  | 14/30 | - |  |
|  | 0 |  |  | 4/15 | 9.1 |  |  | - | 6.3 |  |
|  |  |  |  | 5/15 | 8.1 |  |  | 6/30 | 6.2 |  |
|  |  |  |  | 6/15 | 6.8 |  |  | 7/30 | 6.2 |  |
|  |  |  |  | 7/15 | 5.8 |  |  | 8/30 | 6.0 |  |
|  |  |  |  | 8/15 | 5.3 |  |  | 9/30 | 5.8 |  |
|  |  |  |  | 9/15 | 5.1 |  |  | 10/30 | 5.5 |  |
|  |  |  |  |  |  |  |  | 11/30 | 5.2 |  |
|  |  |  |  |  |  |  |  | 12/30 | 4.8 |  |
|  |  |  |  |  |  |  |  | 13/30 | 4.0 |  |
|  |  |  |  |  |  |  |  | 14/30 | - |  |
|  |  |  |  |  |  |  |  |  |  |  |
